# Climate change impacts on temperate fruit and nut production: a systematic review

**DOI:** 10.3389/fpls.2024.1352169

**Published:** 2024-03-18

**Authors:** Juliana Osorio-Marín, Eduardo Fernandez, Lorena Vieli, Alejandra Ribera, Eike Luedeling, Nicolas Cobo

**Affiliations:** ^1^ Centro de Fruticultura, Facultad de Ciencias Agropecuarias y Medioambiente, Universidad de La Frontera, Temuco, Chile; ^2^ Escuela de Agronomía, Pontificia Universidad Católica de Valparaíso, Quillota, Chile; ^3^ Departamento de Ciencias Agronómicas y Recursos Naturales, Facultad de Ciencias Agropecuarias y Medioambiente, Universidad de La Frontera, Temuco, Chile; ^4^ Departamento de Producción Agropecuaria, Facultad de Ciencias Agropecuarias y Medioambiente, Universidad de la Frontera, Temuco, Chile; ^5^ Department of Horticultural Sciences, University of Bonn, Bonn, Germany; ^6^ Facultad de Ciencias Agropecuarias y Medioambiente, Universidad de La Frontera, Temuco, Chile

**Keywords:** global warming, deciduous trees, warm temperate climate, adaptation strategies, sustainable agriculture, production systems, prediction models, fruit breeding

## Abstract

Temperate fruit and nut crops require distinctive cold and warm seasons to meet their physiological requirements and progress through their phenological stages. Consequently, they have been traditionally cultivated in warm temperate climate regions characterized by dry-summer and wet-winter seasons. However, fruit and nut production in these areas faces new challenging conditions due to increasingly severe and erratic weather patterns caused by climate change. This review represents an effort towards identifying the current state of knowledge, key challenges, and gaps that emerge from studies of climate change effects on fruit and nut crops produced in warm temperate climates. Following the PRISMA methodology for systematic reviews, we analyzed 403 articles published between 2000 and 2023 that met the defined eligibility criteria. A 44-fold increase in the number of publications during the last two decades reflects a growing interest in research related to both a better understanding of the effects of climate anomalies on temperate fruit and nut production and the need to find strategies that allow this industry to adapt to current and future weather conditions while reducing its environmental impacts. In an extended analysis beyond the scope of the systematic review methodology, we classified the literature into six main areas of research, including responses to environmental conditions, water management, sustainable agriculture, breeding and genetics, prediction models, and production systems. Given the rapid expansion of climate change-related literature, our analysis provides valuable information for researchers, as it can help them identify aspects that are well understood, topics that remain unexplored, and urgent questions that need to be addressed in the future.

## 1 Introduction

Most temperate fruit and nut orchards are located at mid-latitudes between 30° and 50°, in both the Northern and Southern hemispheres (Rai et al., 2015). Most of these regions feature a warm temperate climate, which, according to the Köppen-Geiger classification system, is characterized primarily by Mediterranean-like climatic conditions with distinct dry-summer and wet-winter seasons (Kottek et al., 2006; Peel et al., 2007; Sarricolea et al., 2017). Globally, warm temperate climate regions represent 13.4% of the land area (Peel et al., 2007). The climate of these regions is well-suited for temperate fruit crops, which require, among other factors, distinctive cold and warm seasons to meet their physiological requirements, progress regularly through their phenological stages, and achieve secure and sustainable production (Luedeling, 2012; Rodriguez et al., 2021). Throughout the warm temperate zone, temperate fruit crops generate essential revenue for farmers and rural communities.

Overwhelming scientific evidence indicates that recent climate change is attributable to human activity (Rai et al., 2015; Salama et al., 2021; Shukla et al., 2022). Climate change has already affected many natural ecosystems, and it threatens the stability of crop production worldwide. Anthropogenic greenhouse gas emissions have risen dramatically since the mid-20^th^ century, driven largely by economic and population growth. The resulting increases in atmospheric concentrations of carbon dioxide, methane, and nitrous oxide are widely considered the dominant cause of increased temperatures and many extreme weather events during the last century. Future climate change is likely to cause particularly severe impacts in mid-latitude areas, which are expected to be increasingly affected by precipitation irregularities, temperature increases, and prolonged droughts (Parajuli et al., 2019; Shukla et al., 2022). These phenomena are expected to change land suitability for agricultural activities, cause variation in crop growth and development, and challenge the reliability and stability of agricultural production (Bruinsma, 2003). In some areas of the world, mainly in the tropics, increased precipitation frequency and intensity have caused devastating floods, while mid-latitudes are experiencing a decrease in the amount and frequency of precipitation (Bruinsma, 2003), leading to a rising risk of drought (Shukla et al., 2022). The instability of precipitation patterns and changes in local weather conditions are not only detrimental to crop production and quality but they are also generating social and economic uncertainties (del Pozo et al., 2019).

In addition to drought, fruit and nut crops produced in warm temperate regions are strongly threatened by temperature increases during winter, which may affect plant physiology and phenology by compromising the accumulation of winter chill, which is required for important events such as dormancy release (Luedeling, 2012; Rai et al., 2015; Rodriguez et al., 2021). The timing of dormancy release is crucial for fruit producers because, through its impact on flowering times, it determines both the amount of heat the trees can accumulate during the growing season and the risk of exposure to damaging spring frosts, which have frequently affected important growing regions in recent years (Luedeling, 2012).

Because of changing and often erratic weather patterns, agricultural production is facing increasing challenges in places where growing conditions have historically been favorable. While a number of adaptation and mitigation strategies to cope with the effects of climate change on agriculture are being explored by scientists in warm temperate regions, many of these strategies may take as long as 10 to 20 years to be implemented (Bruinsma, 2003). Promising strategies may include shifts to more suitable cultivation areas (Drogoudi et al., 2020; Fernandez et al., 2020a; del Barrio et al., 2021; Rojas et al., 2021; Noorazar et al., 2022; Meza et al., 2023), crop replacement and diversification (Valverde et al., 2015; Almagro et al., 2023; Rezgui et al., 2024), the introduction of new cultivars (Funes et al., 2016; Ruiz et al., 2019; Arenas-Castro et al., 2020; Cantin et al., 2020; Drogoudi et al., 2020), water management strategies (Fraga et al., 2020; Aguirre-Garcia et al., 2021; Gutierrez-Gamboa et al., 2022; Lulane et al., 2022; Espinoza-Meza et al., 2023; Rojano-Cruz et al., 2023), and technological adaptation (Luzio et al., 2021; Mazis et al., 2021; Rojas et al., 2021; Teker, 2023). However, as the impacts of climate change become increasingly severe, the adaptation and adoption of such strategies will require local and regional research, as well as effective governmental policies focused on the implementation of sustainable management practices and the adoption of new technologies. Consequently, it is key for scientists to identify where the current research efforts lie, and what the main knowledge gaps are.

The number of scientific publications on the effects of climate change on agricultural production has been increasing in recent decades, making it difficult for scientists to keep up with current studies and publications. Systematic reviews constitute an effective and reproducible approach to reviewing the existing literature, which allows for unbiased evidence-based conclusions (Koutsos et al., 2019). We conducted a systematic review aimed to identify the current state of the art in research, key challenges and gaps that emerge from studies of climate change effects on fruit and nut crops produced in warm temperate climates. Furthermore, along with the results obtained through the systematic review, we conducted an extended analysis beyond the scope of a regular systematic review. We identified six key research areas and grouped articles according to this classification to gauge the relative importance of these lines of research.

## 2 Methods

To summarize the state of knowledge, we followed the guidelines for conducting systematic reviews in agricultural sciences published by Koutsos et al. (2019), with some adjustments to fit the needs of the present work. The method we used involved six steps: 1) Scoping, 2) Planning, 3) Identification, 4) Screening, 5) Eligibility Assessment, and 6) Interpretation of Results.

The following structured questions were specified prior to the review work and further kept in mind for the development of the manuscript: 1) What are the main topics assessed in studies of climate change effects on temperate fruit and nut crop production in warm temperate climate regions? 2) What strategies have been studied to deal with climate change in temperate fruit and nut crops? and 3) Which are the most studied species in relation to climate change in warm temperate climate regions? To the best of our knowledge, no systematic reviews with the same scope and focused on the questions we addressed here have been published to date.

To carry out the literature review, we conducted a Web of Science (WoS) basic search along with a general Google Scholar search to identify studies on the effects of climate change on fruit and nut production. We identified publications that appeared particularly relevant to the objective of our study and used these studies to build a list of keywords. To identify a suitable search query for conducting this review, a series of searches were conducted in an iterative fashion between January 21^st^ and February 10^th^, 2021, using the advanced search option in the WoS Core Collection database and focusing on studies that were published between 2000 and 2021. Searches were based on a three-section examination query, with sections separated by the Boolean operator “AND”. The first section was aimed at extracting publications on climate change, the second section at extracting publications on fruit and nut production, and section three at placing all publications in warm temperate climate regions. Additionally, within each section several synonyms separated by the Boolean operator “OR” were employed to maximize the capture of publications on each topic. After comparing the results obtained by several search queries, the definitive literature search was conducted on February 10^th^, 2021, using the following query: “TS = (((“global warming”) OR (“climate*”)) AND ((fruit*) OR (“fruit production”) OR (orchards)) AND ((“fruit growing region*”) OR (“warm temperate climate*”) OR (“temperate climate”) OR (“temperate region”) OR (“temperate”) OR (“mediterranean region”) OR (mediterran*) OR ((“wet winter*”) OR (“dry summer*”)))”, where asterisks at end of some words were used as wildcards to represent any number of letters at the end of the word. This query helped us to identify relevant publications to answer the questions addressed in this review. The search was refined to exclude reviews, proceedings, meeting abstracts, book chapters, letters, and editorial material. We complemented the original search, which was performed in early 2021, with an additional search in January 2024 to add the most recent publications.

**Figure 1:**
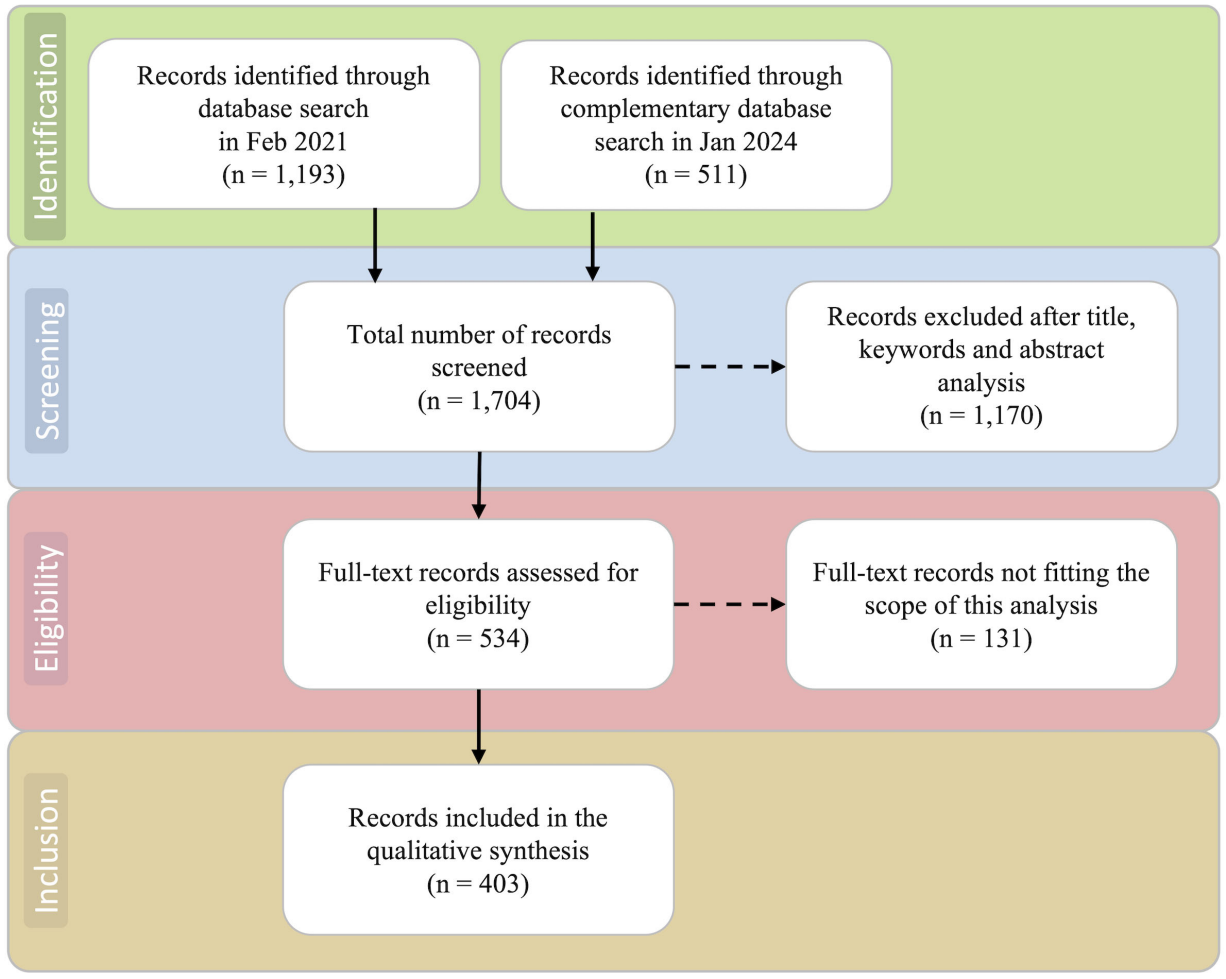
Schematic representation of the workflow adopted for the systematic review process, based on the PRISMA methodology (Moher et al., 2009; Koutsos et al., 2019). Dashed arrows indicate articles that were removed from the analysis. Solid arrows indicate articles retained for the next stage of the analysis.

A database containing all extracted publications was developed in Microsoft Excel for record management and examination (**Figure 1**). Titles, keywords and, whenever necessary, abstracts of all publications were examined to assess the potential relevance regarding the objective of this review. Records that did not fit the scope of our review were excluded from further analyses. Excluded publications focused on crops, products or activities other than temperate fruit species such as tomatoes, soybeans, wheat, cereals, mushrooms, beef, grazing, and foraging, or they featured a geographic scope outside warm temperate climate regions.

With the definitive set of articles established after eligibility assessment, we performed a bibliometric analysis using *biblioshiny*, a web interface for the R package *bibliometrix* (Aria and Cuccurullo, 2017).We used article metadata from the Web of Science database to characterize the literature on the effects of climate change on fruit and nut crops produced in warm temperate climates for number of publications per year, country affiliation of the first author, author collaboration network, international cooperation and crops studied.

Furthermore, in an effort to better understand the results of our search, we manually characterized the literature by assigning a thematic category to each of the analyzed articles and accordingly synthesized the results within six key research areas: (1) *responses to environmental conditions*, (2) *prediction models*, (3) *water management*, (4) *production systems*, (5) *sustainable agriculture*, and (6) *breeding & genetics.* We also defined several sub-topics within each key research area to classify each article according to its research focus. However, given that an article within a major research topic may be relevant for more than one sub-topic, we do not provide a count of articles for each sub-topic (see **Supplementary Table 1** for the complete list of articles classified by main research area).

## 3 Results

Using the definitive search query in February 2021, we identified 1,193 articles in the WoS Core Collection database. Using the same query on January 2024, we identified 511 additional articles published between 2021 and 2023. The resulting dataset consisted of 1,704 records which were further screened for eligibility. After a first evaluation, 1,170 records were excluded because they did not fit the scope of the current review. Only the remaining 534 studies assessing the effects of climate change on temperate fruit and nut production in warm temperate climates were retained.

Further manual curation allowed the detection and removal of 131 additional studies that were either conducted on crops other than temperate fruit and nut trees (e.g., avocado, coriander and pine) or not primarily focused on the effects of climate change, adaptation actions or mitigation strategies. After manual data filtering and curation, a final set of 403 articles were retained for detailed examination and used for bibliometric analyses (**Figure 1**; **Supplementary Table 1**).

### 3.1 Bibliometric analysis

**Figure 2:**
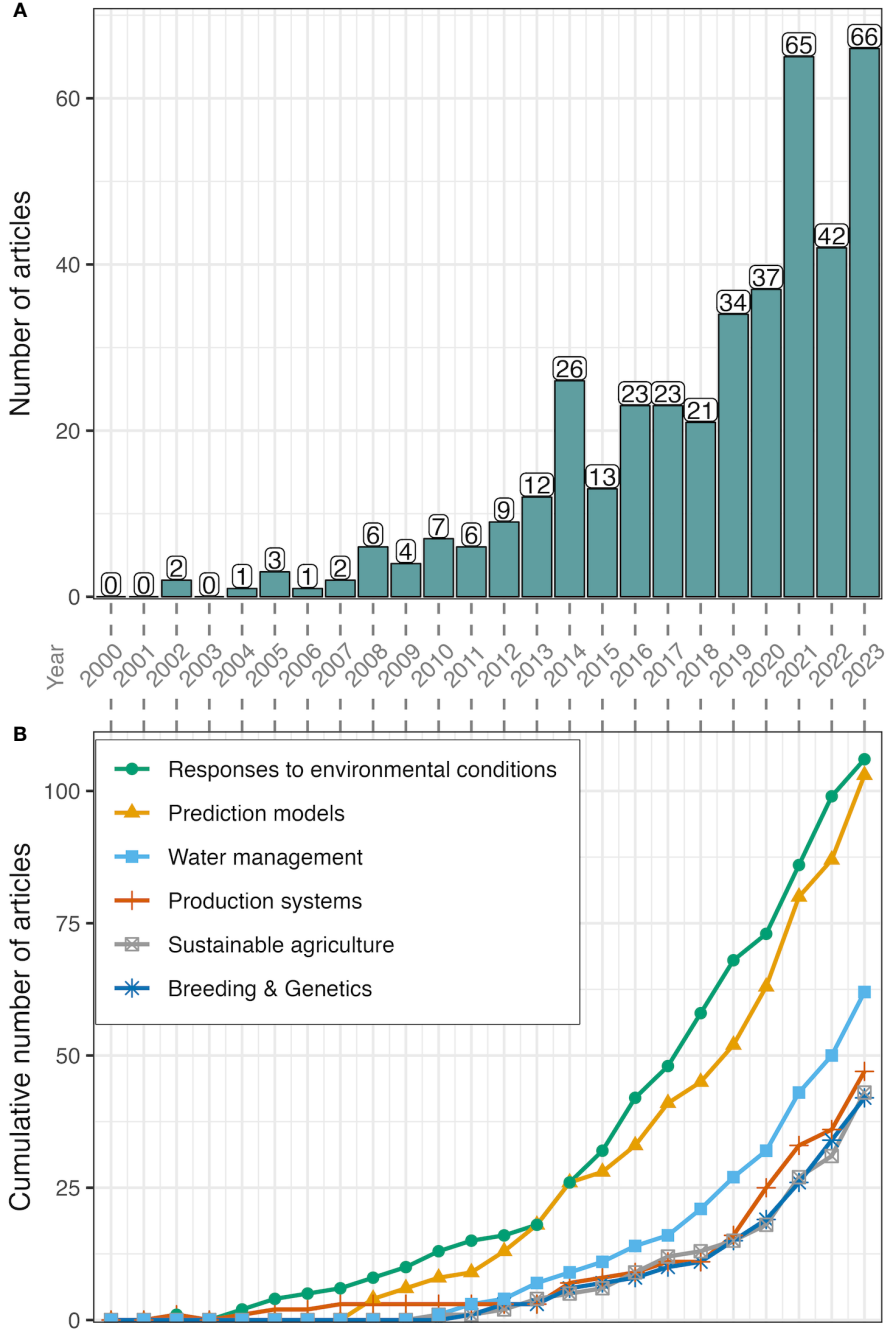
Scientific production by number of articles studying climate change effects on temperate fruits and nuts published by year **(A)** and evolution of key-topics by year **(B)**, between 2000 and 2023. Labels of the x-axis (year) are located in between figure panels.

Annual scientific productivity (i.e., the number of published articles per year) related to the effects of climate change on fruit and nut production in warm temperate climate regions has continuously increased during the last two decades (**Figure 2A**). No articles published in 2000, 2001, and 2003 were identified with this search. In contrast, 66 articles were identified in 2023.

To visualize long-term changes in scientific productivity over time, we divided the timeframe between the years 2000 and 2023 into four six-year periods (2000-2005, 2006-2011, 2012-2017, and 2018-2023). The rising tendency observed in the yearly data became more evident when using the six-year terms, with a dramatic 44-fold difference between the first period (2000-2005: 6 articles) and the last one (2018-2023: 265 articles). Furthermore, although the magnitude of change is smaller among consecutive terms, a consistent > 2.5-fold increase demonstrates that the number of articles has increased steeply since the 2000s (2006-2011: 26 articles; 2012-2017: 106 articles).

**Figure 3:**
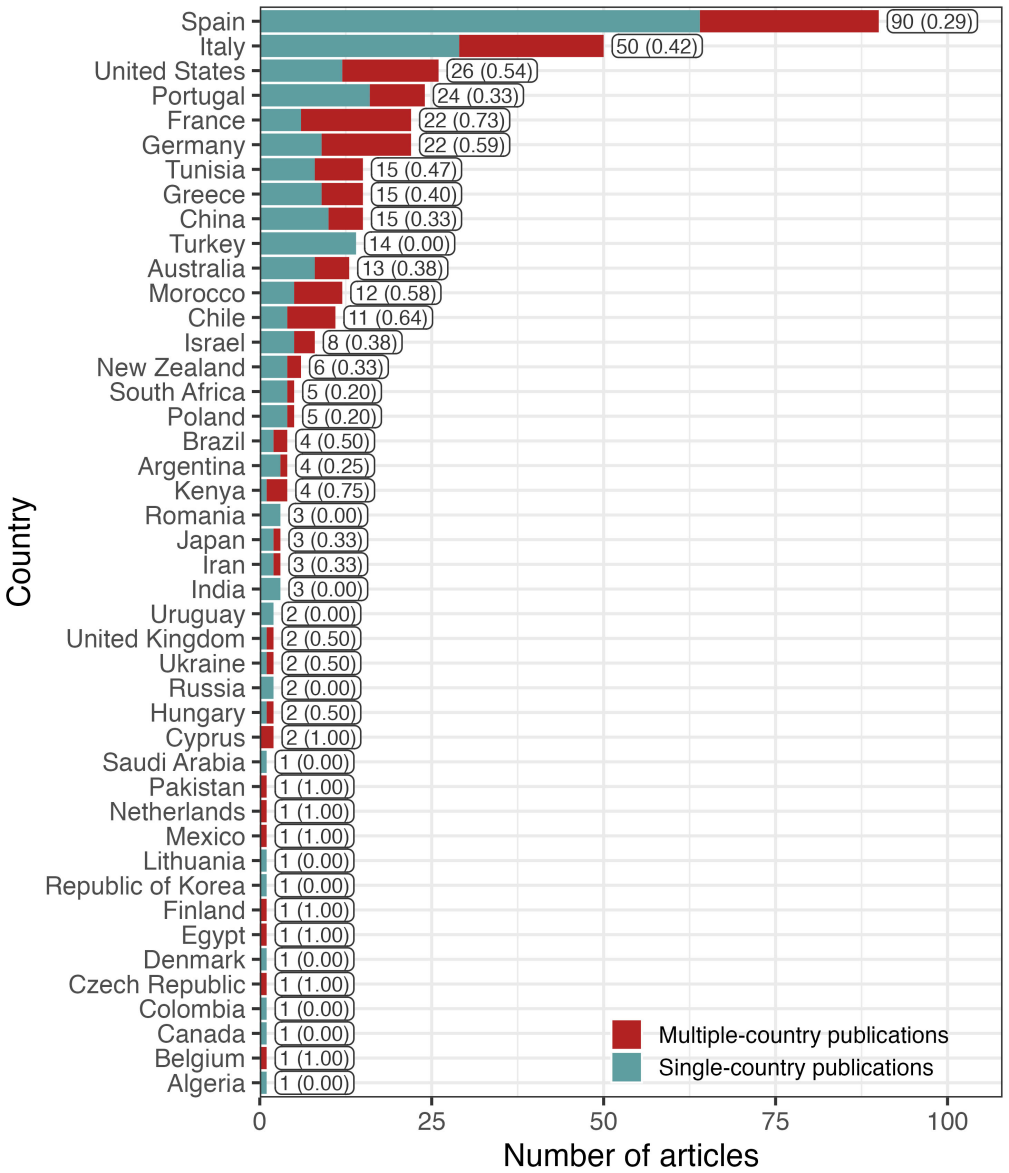
Total number of articles published by country, assessed by corresponding author’s country affiliation. For each country, the number of articles including authors affiliated to more than one nation (i.e., international collaboration) are shown in red and articles published by authors affiliated to a single country in green. Bar labels indicate the total number of articles per country, followed by the proportion of articles with authors affiliated to more than one country in brackets.

We used the affiliations of corresponding authors as a proxy to identify regions of the world that have led scientific research on climate change and its effects on fruit and nut production in temperate regions during the last two decades. We detected a large difference between the number of articles led by authors in the northern and the southern hemispheres. Authors affiliated to institutions located north of the equator led 354 articles, representing 88% of the articles analyzed in this review. Among the countries in the northern hemisphere, Spain (90), Italy (50), USA (26) and Portugal (24) are most frequently represented (**Figure 3**). Conversely, only 49 articles were led by authors based in the southern hemisphere, with most of these authors affiliated with institutions in Australia (13), Chile (11), New Zealand (5) and South Africa (5). International cooperation, assessed by the number of articles with authors affiliated with institutions from more than one country, revealed that research on climate change is a highly collaborative effort, with 40% of the articles written by authors from multiple countries. Spain, Italy, USA, Portugal, Germany, and France were the countries with the highest numbers of published articles (58% of articles), with an average of 48% of them including authors affiliated to more than one country. The multiple-country publication (MCP) ratio (i.e., the proportion of articles including at least one author based in a different country from the corresponding author) shows that the Kenya (MCP ratio: 0.75), Chile (MCP ratio: 0.727), France (MCP ratio: 0.636) and Germany (MCP ratio: 0.591) have the highest proportion of articles with international collaboration (among countries with more than two publications). However, the MCP ratio may not be the best index to compare international collaboration efforts among countries because it is strongly influenced by the total number of publications. In fact, the publication outputs of the six most prolific countries (Spain, Italy, United States, Portugal, France and Germany) include 98 articles with international collaborators, compared to just 64 such publications among the remaining 38 countries (**Figure 3**).

**Figure 4:**
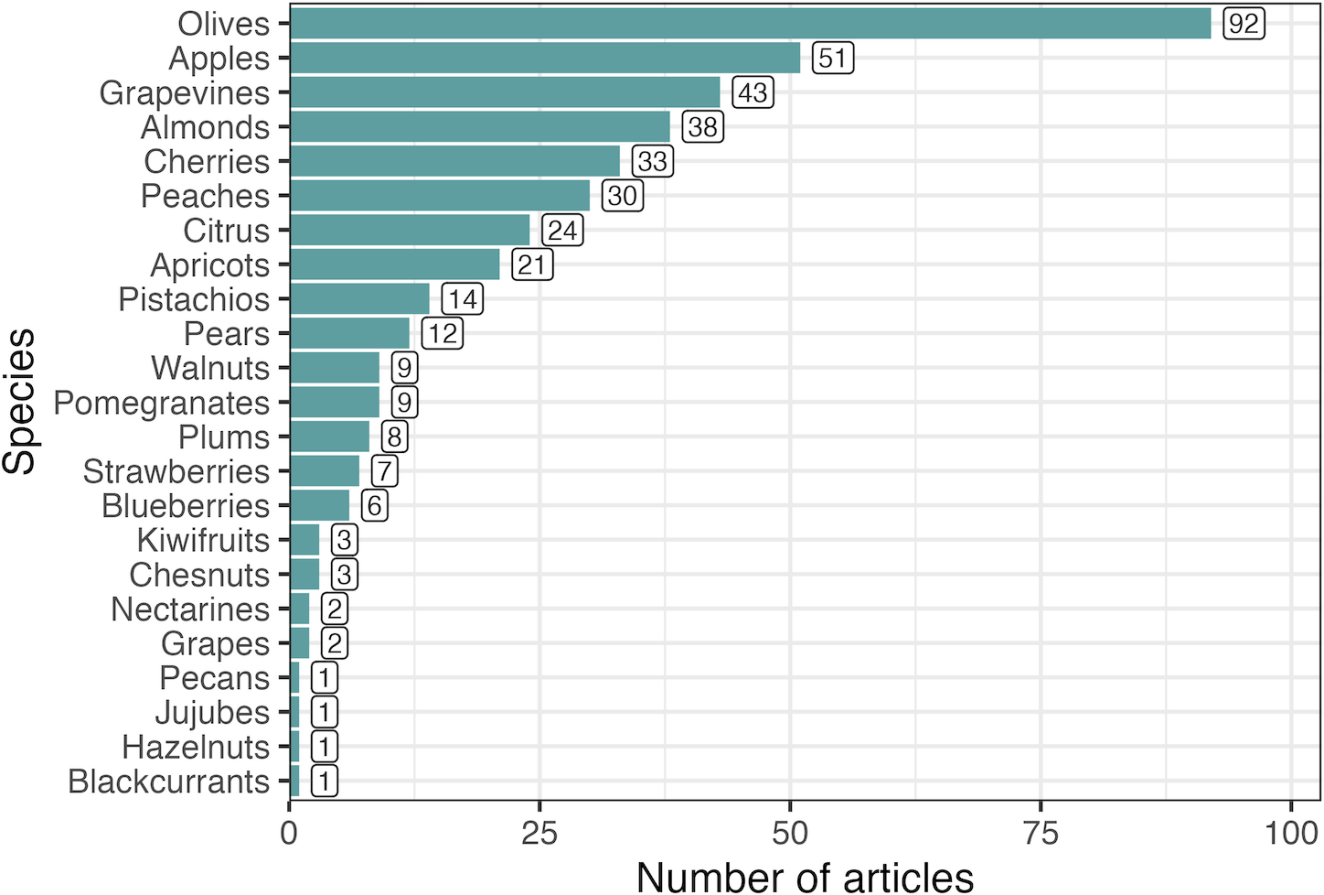
Most studied crops according to a manual classification of articles. Bar labels indicate the total number of articles studying a given crop. Because articles may have studied more than one species, the sum of articles per species is greater than the number of articles analyzed.

Based on a manual classification of articles, the ten most studied crops were olives (*Olea oleracea*, 92), apples (*Malus domestica*, 51), grapevines (*Vitis vinifera*, 43), almonds (*Prunus dulcis*, 38), cherries (*Prunus avium*, 33), peaches (*Prunus persica*, 30), citrus (24), apricots (*Prunus armeniaca*, 21), pistachios (*Pistacia vera*, 14) and pears (*Pyrus communis*, 12) (**Figure 4**). It is important to note that the number of studies focusing on olives and grapevines may be strongly influenced by the historic use of deficit irrigation strategies to manage fruit and end-product quality.

### 3.2 Key research topic analysis

**Figure 5:**
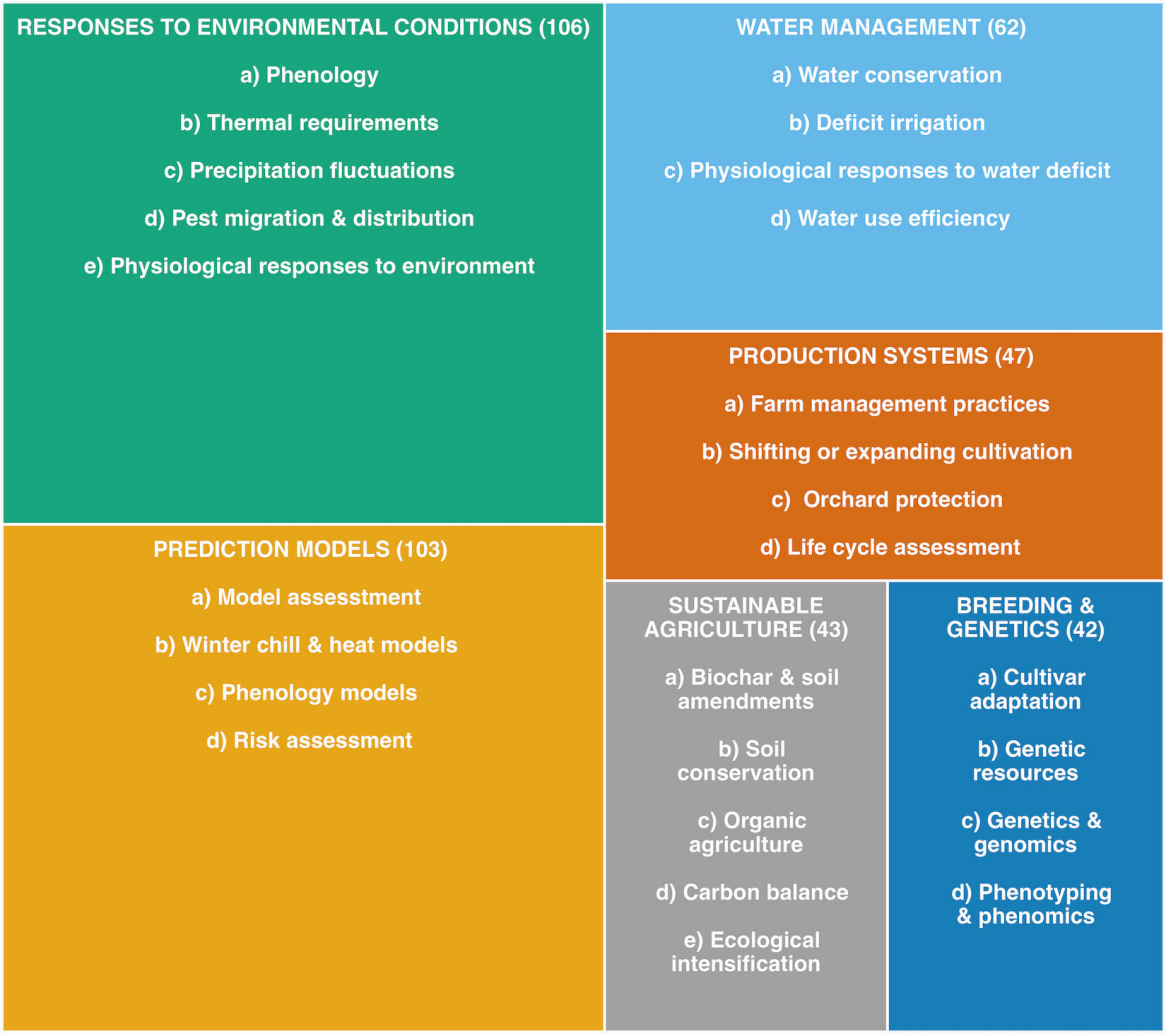
Key research topics and sub-topics identified based on title, abstract and full text of the 403 articles analyzed. Each of the six key research topics is listed in capital letters, and the number of studies classified under them is represented by the area of the rectangles and indicated in parentheses next to each topic. A list of sub-topics identified within each key research topic is presented below each title.

To better understand the research focus of the literature analyzed, we manually categorized the set of 403 records used for bibliometric analyses according to six key research areas, and further divided them into several sub-topics (**Figures 2B**, **5**). The topics and sub-topics proposed in this review are the result of a thorough manual examination. They intend to characterize the main topics studied in the articles evaluated in this review beyond the information extracted from the bibliometric analysis. It is important to note that within each major topic, many articles touched on more than one sub-topic. Although publications often provide analyses and conclusions on more than one major topic, as research in climate change is transversal by nature, here we attempted to provide an overview of each topic to characterize the state of the art (**Supplementary Table 1**).

Research on plant responses to environmental conditions was the most abundant publication category, accounting for 26.3% (106 studies) of the articles included in the analysis. Within this topic, we defined five sub-topics that covered articles related to phenology, thermal requirements (chill/heat), precipitation fluctuations, pest migration and distribution, and physiological responses to the environment. With a similar number of articles, research on prediction models can be grouped into articles on model assessment, winter chill and heat models, phenology models, and risk assessment. Prediction modeling, a statistical approach to predict and forecast future events, has been a trending topic in climate change studies, as evidenced by the high number of publications detected in this review (103 studies, 25.5% of all publications). The third most abundant category grouped articles on water management, with 62 articles (representing 15.4% of the included literature) which focus on topics related to water conservation, deficit irrigation, physiological responses to water deficit, and water use efficiency. Forty-seven articles focused on research related to farm management practices, shifting or expanding cultivation, orchard protection, and life cycle assessment (LCA), representing 11.7% of the articles analyzed here. We identified 43 articles focused on topics related to the sustainable production of fruit and nut crops, accounting for 10.7% of the literature analyzed and reporting research related to the use of biochar or other soil amendments, soil conservation practices, organic agriculture, carbon balance, and ecological intensification. The sixth category, based on the number of publications, grouped articles related to breeding and genetics topics. Forty-two articles focusing on cultivar adaptation, genetic resources, genetics, genomics, and phenomics represent 10.4% of the total articles analyzed.

## 4 Discussion

### 4.1 Bibliometric analysis

Over the last two decades, the effects of climate change have become increasingly evident, with irregular weather patterns and frequent extreme climatic events impacting agriculture worldwide (Cogato et al., 2019). The steady increase in the number of publications on climate change and fruit and nut production in warm temperate climate regions reported in this analysis reflects a growing interest in research related to both a better understanding of the effects of climate anomalies on crops and the need to find strategies that allow this industry to adapt to current and future weather conditions while reducing the environmental impacts of agriculture. Perennial crops, unlike annual crops, require farmers and other stakeholders to make decisions over longer time periods (Lobos and Hancock, 2015; Ahmed et al., 2021; Meza et al., 2023). As a result, there is a need for research that can synthesize evidence on the effects of environmental factors related to climate change, as well as management conditions that are relevant to climate adaptation. This research can then inform climate change mitigation and adaptation strategies in these production systems (Ahmed et al., 2021).

Our analysis revealed a significant difference in scientific productivity between countries, based on the affiliations of the corresponding authors. Although the magnitude of the difference between hemispheres may partly be due to most countries with warm temperate climates being located north of the equator and expending more funding for this type of research, a recent study reported a similar proportion for studies on development and development policy (Amarante et al., 2022).

Authors from four countries led close to half of the articles we analyzed. Furthermore, while 40% of the articles represent international collaboration, we believe that leveraging technical skills and access to research funding and infrastructure, especially in developing nations, should be a priority (Cummings and Hoebink, 2017). For example, because breeding improved cultivars is an expensive long-term endeavor (Meza et al., 2023), developing countries may not have access to the necessary technical and financial resources to support such investment. Accordingly, both local capacity building and international collaboration, with a strong participation of local communities and considering the constraints of local production, could be an effective strategy towards the development of a more adapted agriculture that is resilient to the effects of climate change and remains sustainable over time.

### 4.2 Key research topic analysis

#### 4.2.1 Responses to environmental conditions

Global warming is widely expected to increase the frequency of above-normal temperatures (Legave et al., 2015; Santos et al., 2017). Such temperatures are the main reason for the increase in heat and decrease in winter chill accumulation that have been observed in most places (Campoy et al., 2011; Luedeling, 2012; Atkinson et al., 2013; Lippmann et al., 2019). For temperate fruit and nut trees, adequate winter chill and heat accumulation are critical requirements for satisfactory commercial production (Gordo and Sanz, 2009; Cerutti et al., 2011; Luedeling et al., 2013; Darbyshire et al., 2016; Horikoshi et al., 2018; Fadon et al., 2020; Fernandez et al., 2020a; del Barrio et al., 2022). Insufficient winter chill and increasing heat during key phenological stages can affect important physiological processes, leading to non-uniform flowering (El Yaacoubi et al., 2014; Orlandi et al., 2014; Legave et al., 2015; Funes et al., 2016; Elloumi et al., 2024), delayed bud formation (Bonhomme et al., 2005; El Yaacoubi et al., 2016; Yi et al., 2020; Barba-Espin et al., 2022), abnormal bud-break (Bonhomme et al., 2005), fruit set reduction (Hedhly et al., 2007; Ruiz and Egea, 2008; Benlloch-Gonzalez et al., 2018; Egea et al., 2022), and ultimately reduced yield potential (Benmoussa et al., 2018; Moriondo et al., 2019; Mujahid et al., 2020; Fernandez et al., 2021). The literature reports a consistent decline in winter chill in all warm temperate climate regions around the world, which explains the high research interest in this topic. Since winter chill and heat accumulation are required for temperate fruit and nut crops to break dormancy, choosing appropriate cultivars for a given region is crucial to ensuring orchard productivity.

As temperature increases, prolonged drought events and variable precipitation patterns are becoming a major concern since they can cause intense drying and a reduction of irrigation water resources (Ponti et al., 2009; Gargouri et al., 2012; Ronco et al., 2017; Silva et al., 2018). Increased exposure of flowering trees to spring frost damage has also been studied, since early flowering due to increased winter temperature may expose flowers to spring frost events (Blanke and Kunz, 2009; Eccel et al., 2009; Matzneller et al., 2016; Leolini et al., 2018; Rojas et al., 2021; Yang et al., 2021; Fernandez et al., 2023). To avoid spring frost damage during the flowering period, many agricultural systems include artificial roof systems, wind machines or water sprinkler systems for protection (Ohata et al., 2017), or rely on late-blooming cultivars (Guo et al., 2019; Liu et al., 2021; Sapkota et al., 2021). Some other strategies, including the use of low-chill cultivars, dormancy avoidance, microclimate manipulation, and rest-breaking chemicals, have also been implemented to produce temperate fruit and nut crops in warm regions (Rai et al., 2015; Fernandez et al., 2023; Meza et al., 2023). Because of changing weather patterns, several studies have evaluated the capacity of crops to perform in regions that are becoming suitable for agriculture (del Barrio et al., 2021, del Barrio et al., 2022; Meza et al., 2023). However, because of the climatic variability across the world, it is imperative that further research is conducted at the local level to ensure the selection of appropriate cultivars (refer to Luedeling, 2012 for a detailed explanation).

Insect and pathogen communities are greatly affected by temperature fluctuations and climatic events. We identified several publications dedicated to studying pest migration, distribution, and adaptation, along with the risk of emerging pest threats in current and new cultivation areas due to warming weather (Gutierrez et al., 2008; Nyamukondiwa and Terblanche, 2010; Sanders et al., 2010; Sharma, 2014; Serga et al., 2015; Stephens et al., 2016; Costi et al., 2017; Gutierrez et al., 2018; Weldon et al., 2018; Qin et al., 2019; Gutierrez et al., 2021; Bogdanovich et al., 2023). Most of these articles project the future distribution of pests or changes in habitat suitability due to rising temperatures. For example, as mild winters become more frequent, effective reduction of insect populations due to cold temperature is compromised and therefore pest pressure may increase over time (Lippmann et al., 2019; Gutierrez et al., 2021). Furthermore, as spring and summer seasons become warmer, insect reproduction rates will likely increase, increasing population size, raising the pests’ ability to adapt to new scenarios, and exerting increasing pressure on host crops (Luedeling et al., 2011; Lippmann et al., 2019).

#### 4.2.2 Prediction models

These studies have allowed the development of agroclimatic forecasts for a range of future scenarios, such as the Representative Concentration Pathways (RCPs) proposed by the IPCC, which are used to describe different climate outcomes under a range of climate forcing scenarios. For instance, winter chill and heat models, essential for quantifying winter chill and heat requirements, are fundamental for facilitating the selection of appropriate cultivars (Luedeling et al., 2009a; Luedeling et al. 2009b; Luedeling et al., 2009c; Luedeling and Gassner, 2012; Benmoussa et al., 2018; Delgado et al., 2021; Fernandez et al., 2023; Meza et al., 2023). They have been evaluated in different scenarios to determine the likelihood that a region or location can exploit new opportunities for establishing temperate orchards (Sahu et al., 2020; del Barrio et al., 2021), or to identify agricultural adaptation strategies (Cabezas et al., 2020; Meza et al., 2023).

Nevertheless, according to the reviewed literature, many of the existing models deliver questionable projections when used to predict winter chill accumulation for climate change scenarios. Models vary greatly in their sensitivity to warming (Luedeling et al., 2009b), which leads to inconsistent projections when using models across multiple locations that feature differences in climate (Fernandez et al., 2020b, Fernandez et al., 2023). Luedeling (2012) explains that several winter chill models are entirely based on empirical observations, mainly collected in the location where they were developed, leaving observed phenological variation of bloom dates across locations unexplained. Therefore, the prediction capacity of winter chill models should be questioned, and predictions should be interpreted with caution, especially given recent and projected climate change. Several authors suggest that research on prediction models and forecasting should focus on the development of updated models to ensure appropriate heat and chill quantification, helping farmers prepare for the upcoming effects of climate change (Luedeling et al., 2009b; Luedeling, 2012; Elloumi et al., 2013; Darbyshire et al., 2014; Luedeling, 2018; Campoy et al., 2019; Parkes et al., 2020; Meza et al., 2023).

Several publications included in this review focus on risk assessment analysis to comprehend the scope of climate change effects. These studies are diverse in nature, providing outcomes and guidelines for the potential risk of flood damage (Kourgialas and Karatzas, 2016), desertification and drought (Gargouri et al., 2012), water scarcity (Ronco et al., 2017; Arenas-Castro et al., 2020), and yield potential (Fraga et al., 2020), among other factors. Having the ability to predict the future through models greatly enhances our ability to study and understand prospective climate outcomes, and to develop adequate responses to mitigate or adapt to the predicted conditions. Risk assessments have also been used to evaluate the likelihood of a cultivar meeting its winter chill requirements (Measham et al., 2014; Darbyshire et al., 2016; Fernandez et al., 2020a), and to provide relevant data through a platform targeting growers and researchers to support strategic orchard management decisions (Pathak et al., 2018; Fernandez et al., 2022). Accurate prediction models can provide crucial insights to policymakers and stakeholders to develop and implement adaptation strategies that help secure the sustainability of the fruit industry under future scenarios.

#### 4.2.3 Water management

Water scarcity, aggravated by the effects of climate change, is predicted to worsen due to more severe and frequent drought events in warm temperate climate regions (Ronco et al., 2017; Galindo et al., 2018; Pedrero et al., 2020). This will decrease land suitability for fruit crops in many regions, especially in arid and semi-arid areas where rainfall is becoming less intense and frequent (Ronchail et al., 2014; Galindo et al., 2018; Araya-Osses et al., 2020; El-Otmani et al., 2020; Meza et al., 2023). Increasing annual mean temperature due to climate change also threatens the availability of freshwater. Along with reductions in water availability, higher temperatures increase evapotranspiration and crop water demand (Rai et al., 2015; del Pozo et al., 2019; Arenas-Castro et al., 2020; Fraga et al., 2020). The aforementioned conditions may explain the strong focus on water-saving strategies, such as water deficit irrigation management, that we observed in our analysis.

Deficit irrigation has historically been the main strategy used in olive trees and grapevine to manage fruit quality (Fernandes de Oliveira et al., 2013; Galindo et al., 2018; Araujo et al., 2019; Buesa et al., 2019; Kokkotos et al., 2020; Siakou et al., 2021; Ibba et al., 2023; Ferrara et al., 2024), and it is becoming a popular strategy in other crops, including mandarins (El-Otmani et al., 2020), pomegranates (Galindo et al., 2018; Adiba et al., 2022), apples (Panzacchi et al., 2012; Kendall et al., 2022), almonds (Gutierrez-Gordillo et al., 2020; Barreales et al., 2023), peaches (Ruiz-Sánchez et al., 2018), apricots (Ezzat et al., 2021), and pistachios (Marino et al., 2018). In brief, the idea is to reduce the amount of water provided to the crop during the growing season, improving marketable yield per unit of water and end-product quality, rather than achieving maximum yields (Ronco et al., 2017; Galindo et al., 2018; El-Otmani et al., 2020). However, some physiological stages such as blooming and fruit or nut filling are very sensitive to water deficit. Hence, deficit irrigation strategies need to be tailored for the specific needs of each crop, to maximize water savings without compromising fruit quality and yield (Galindo et al., 2018; Ruiz-Sánchez et al., 2018). A deficit irrigation strategy that is carefully adjusted to crop needs during all phenological stages can maximize water savings, without impacting fruit size and quality (Ruiz-Sánchez et al., 2018). Galindo et al. (2018) provide a complete review of water deficit strategies, proposing adoption of crops that are able to withstand low water supply, and highlighting the need for research on the risk of soil salinization, which may result from such irrigation strategies.

Two other strategies for water-savings that were mentioned in the reviewed literature are the use of desalinated water (Martin-Gorriz et al., 2014; Romero-Trigueros et al., 2021) and wastewater for irrigation purposes (Moretti et al., 2019; Kokkotos et al., 2020). Nonetheless, Martin-Gorriz et al. (2014) state that, while possibly conferring some adaptation benefits to fruit producers, using desalinated water may also accelerate global warming, since water desalination is an energy-intensive process. However, other authors propose that as water desalination is becoming cheaper and more sustainable, more research on the safe use of desalinated water in agriculture is needed (Aldaya et al., 2019). Besides desalinated water, wastewater is a plausible source for supplementing irrigation water in regions where drought is limiting food production (Moretti et al., 2019; Pedrero et al., 2020). Wastewater may come from different sources such as municipal effluents, agriculture, animal production and industrial processes (Pedrero et al., 2020). Nevertheless, in addition to the treatment required to safely use wastewater for irrigation in agriculture (Meli et al., 2002; Pedrero et al., 2020), some parameters such as salinity, sodicity, metal and trace elements, and organic materials may need to be closely monitored and controlled to ensure the safety of using such water resources for crop irrigation (Moretti et al., 2019; Pedrero et al., 2020).

Since water is essential for basic physiological processes, several studies have focused on the physiological responses of trees to water stress (Panzacchi et al., 2012; Zapata-Sierra and Manzano-Agugliaro, 2017; Torres et al., 2018; Gutierrez-Gordillo et al., 2020; Adiba et al., 2021). Examples of such processes and phenomena whose responses to water stress have received scientific attention are berry composition in grapevines (Fernandes de Oliveira et al., 2013; Permanhani et al., 2016; Buesa et al., 2019), anthocyanin levels in grapes (Fernandes de Oliveira et al., 2013), fruit quality in citrus and pistachios (Garcia-Tejero et al., 2011; Marino et al., 2018), phenolic composition in sweet cherries (Blanco et al., 2020), biomass in olives (Viola et al., 2012, Viola et al., 2014; Pierantozzi et al., 2020) and yield increase or decrease in grapes, olives and pistachios (Garcia-Tejero et al., 2011; Viola et al., 2012; Permanhani et al., 2016; Blanco et al., 2020; Pierantozzi et al., 2020). Most of these studies were aimed at increasing fruit quality and crop sustainability under water scarcity.

In some countries of the warm temperate climate region, governments are promoting or investing in irrigation infrastructure and encouraging farmers to adopt new technologies for sustainable irrigation (Roco et al., 2014; Galindo et al., 2018). However, such adaptation actions can be difficult to take. In Chile, for instance, farm-level adaptation is lagging behind scientific knowledge and government policies, mainly because of farmers’ highly variable socio-economic conditions and the high level of investment required to establish such infrastructure (Roco et al., 2014). Nonetheless, farmers’ ability to strategically manage water resources and to attune water availability to the needs of each specific crop and cultivar may make a difference in preserving freshwater for future generations.

#### 4.2.4 Production systems

Weather instabilities along with extreme climatic events have forced growers to look for agricultural practices that can help them adapt to the changing climatic regime. Rest-breaking chemicals have often been used to compensate for the lack of chill accumulation caused by increased temperatures (Gariglio et al., 2012; Ghrab and Ben Mimoun, 2014). As an alternative to chemical treatments, Mujahid et al. (2020) proposed using a cold plasma treatment to break dormancy in grapes. Beyond chill and dormancy control, optimizing nutrient use efficiency (Swarts et al., 2016), irrigation scheduling and harvest timing (Paltineanu and Chitu, 2020) are other farm management practices mentioned in the literature that can be used to reduce greenhouse gas emissions and facilitate climate change mitigation.

Microclimate management, such as water supply management or hail and shade nets, as well as soil management, such as tilling or mulching, can be effectively used in production systems to help reduce agriculture’s pressure on the environment (Houston et al., 2018; Campi et al., 2020). Protective covers and canopy management techniques are often used to mitigate the effects of climate change and have allowed for the expansion of agricultural production to new production areas. For instance, the use of polyethylene covers was evaluated in sweet cherry orchards in Chile, where such protective measures may provide protection from rain, hailstorms, and extreme cold temperatures as a strategy to expand production to new potential growing regions (Blanco et al., 2021; Rojas et al., 2021).

Along the same lines, another promising strategy identified in the literature to reduce the risk of insufficient winter chill accumulation or escape spring frost damage and desertification is the redistribution, shifting or expansion of cultivation to regions that may become favorable for fruit crop production, leading to the reconfiguration of agricultural landscapes (Ponti et al., 2009; Luedeling et al., 2009a; Dag et al., 2014; Tanasijevic et al., 2014; Thomson et al., 2014; Ohata et al., 2017; Rana et al., 2017; Hepaksoy et al., 2018; Leolini et al., 2018; Guo et al., 2019; Ben Rouina et al., 2020; Sahu et al., 2020; del Barrio et al., 2021; Rodriguez et al., 2021; Meza et al., 2023). The main limitation to most of these studies, however, is that they are solely based on the configuration of bioclimatic conditions, without considering local agricultural land use distribution, competition between land uses, and the adaptive capacity of crops (Houston et al., 2018; Sahu et al., 2020; del Barrio et al., 2021). A study in Argentinian Patagonia concluded that the increasing heat availability in the south of the southern hemisphere presents an opportunity for fruit and nut growers since new species and cultivars with temperate-climate requirements can be introduced into new regions outside their traditional ranges (del Barrio et al., 2021, del Barrio et al., 2022). Other studies evaluated the performance of olives in hot desertic areas to establish their yield potential and olive oil quality for future production (Dag et al., 2014; Ben Rouina et al., 2020).

Different strategies have also been proposed to protect crops from rising temperature and diminish sunburn damage. Excess heat and the additional water stress that it causes, can lead to a decrease in growth, leaf function, productivity, and fruit quality. Therefore, it is important to ensure that adequate measures are in place to mitigate the impact of these factors on plant health and overall performance. White kaolin powder sprays, clay, calcium carbonate and wax emulsion sprays have been used to reduce radiation load by increasing canopy albedo (Rosati et al., 2007; Houston et al., 2018; Meza et al., 2023; Teker, 2023). Other methods proposed to reduce radiation include leaf canopy management to keep the fruit shaded (Feng et al., 2018; Garrido et al., 2018; Martínez-Lüscher et al., 2020; Meza et al., 2023) and the installation of shading nets covering orchards and vineyards (Lobos et al., 2012; Feng et al., 2018; Mupambi et al., 2018). Besides methods oriented to reduce radiation, studies support the use of overhead irrigation misting systems to avoid heat stress in mid-summer (Kliewer and Schultz, 1973; Feng et al., 2018; Martínez-Lüscher et al., 2020).

#### 4.2.5 Sustainable agriculture

According to the Intergovernmental Panel on Climate Change (Shukla et al., 2022), the agriculture, forestry, and other land uses (AFOLU) sector was responsible for 13 - 21% of direct anthropogenic greenhouse gas (GHG) emissions between 2010 and 2019. On the other hand, agriculture is also responsible for considerable carbon sequestration through soil and plant activity, which contributes to mitigating climate change (Aguilera et al., 2015; Mohamad et al., 2016; Almagro et al., 2017; Shukla et al., 2022).

Intensive agricultural farming systems require a lot of energy for food production (Mazis et al., 2021). Conventional farming systems coupled with climate change often lead to the reduction of soil organic matter, soil erosion, desertification, and degradation of water resources (Michalopoulos et al., 2020; Niu et al., 2021), leading to less carbon storage in those systems. However, research in olive orchards supports that intensification can improve carbon sequestration and soil quality (Mairech et al., 2020, Mairech et al., 2021; Taguas et al., 2021). Although sustainable agricultural practices have been applied for a long time, as the effects of climate change become more evident, alternative farming practices have recently gained new prominence due to conducive policies and increased consumer demand for fruits and nuts with low environmental impact.

Sustainable practices, such as minimum or no tillage (Cook, 2006; Rodriguez-Pleguezuelo et al., 2017), weed mowing (Martin-Gorriz et al., 2020), cover cropping (Marquez-Garcia et al., 2013; Wolf et al., 2017), incorporation of biochar and organic soil amendments (Stavi, 2013; Baronti et al., 2014; Walkiewicz et al., 2020), biomass accumulation and crop diversification (Martin-Gorriz et al., 2020; Michalopoulos et al., 2020), are potentially beneficial measures to offset anthropogenic greenhouse gas emissions (Stavi, 2013) and provide efficient mechanisms against soil degradation and desertification (Martin-Gorriz et al., 2020). However, none of these practices alone consistently leads to positive effects on ecosystems (Kleijn et al., 2019a). More studies on sustainable practices may be needed at the regional level to help reduce knowledge gaps related to local farming practices and to help farmers make informed decisions.

Organic agriculture has been proposed and evaluated as an adaptation strategy to help reduce the impacts of climate change (Mohamad et al., 2016). Granatstein et al. (2016) reported that organic agriculture has grown by 109% in Europe and North America for organic temperate fruit trees between 2008 and 2013. Some benefits derived from organic farm management include increased carbon sequestration, reduced erosion, soil fertility restoration and biomass accumulation (Martin-Gorriz et al., 2020). However, Mohamad et al. (2016) found that the use of manure for fertilization in organic agriculture increased GHG emissions, even though an increase in soil carbon content compensated, at least partially, for the negative effect of these emissions.

Ecological intensification is based on the assumption that the delivery of ecosystem services is suboptimal in high-input agricultural systems (Bommarco et al., 2013). This production strategy has been proposed as an alternative to maximize productivity while minimizing potential negative environmental effects, by harnessing ecosystem services to complement or substitute external inputs, such as synthetic fertilizers and agrochemicals (Bommarco et al., 2013; Kleijn et al., 2019b). Despite the use of wildflower strips having become a popular strategy in temperate orchard systems, their implementation in Mediterranean orchard systems remains understudied (Mockford et al., 2023). In a recent study, Mockford et al. (2023) evaluated the suitability of 12 native perennial species for wildflower strips in commercial citrus orchards over a three-year period, reporting increasing plant species richness and greater availability of resources expected to support natural enemies. Agroforestry is a model of ecological intensification that has the potential to provide multiple ecosystem services and contribute to biodiversity conservation in agricultural landscapes (Torralba et al., 2016; Panozzo et al., 2022). These systems have been associated with improvements in crop growth and yield, particularly in tropical and subtropical regions (Jose, 2009; Tscharntke et al., 2011; Mbow et al., 2014). In the Mediterranean area, where the risk of yield losses due to climate issues is increasing, the impact of trees on the microclimate and edaphic environment might be beneficial for understory crops (Panozzo et al., 2022). Although agroforestry has a long tradition in the Mediterranean region dating back to pre-Roman times, due to agricultural intensification, traditional agroforestry systems have not been implemented in the last few decades in a large part of this area (Panozzo et al., 2022; Rezgui et al., 2024). We identified four studies published recently focusing on the implementation of agroforestry systems to enhance farm sustainability and diversification (Bateni et al., 2021; Zahoor et al., 2021; Panozzo et al., 2022; Rezgui et al., 2024).

Carbon sequestration has also been studied as a mitigation measure to restore degraded land. Temperate fruit orchards and vineyards are currently receiving much attention for their potential to act as carbon sinks to help raise soil organic matter contents (Phani Kumar et al., 2010; Scandellari et al., 2016; Mairech et al., 2020; Mairech et al., 2021; Taguas et al., 2021). Montanaro et al. (2017) studied carbon budgets in peaches with the purpose of establishing the best soil management method to improve carbon sequestration and raise soil organic carbon content. Overall, the research we reviewed indicates that advances towards sustainable and climate-smart fruit production can be achieved through a range of practices, including improving soil management, incorporating crop residues and reducing tillage.

It is important to highlight that major advances towards sustainable agricultural production across the globe seem unlikely without changes in policies, economic incentives and farm adaptation (Shukla et al., 2022). Such measures need to consider the costs and benefits for farmers, including the impact on crop yield stability. Therefore, most authors agree that adequate strategies for sustainable farm management need to be implemented at local level in order to reduce GHG emissions. Sustainable farming practices that combine elements of conventional and organic practices may hold promise for reducing the negative impact of agricultural practices on the environment (Ceccanti et al., 2020).

#### 4.2.6 Breeding and genetics

Genetic improvement is a long-term strategy that involves the development of new, adapted cultivars that are able to withstand the adversities of climate change (Iwata et al., 2016; Houston et al., 2018). To improve knowledge and enhance the capacity to react to changing climate conditions, breeders should have access to genetic diversity and genetic resources that thrive under marginal growing conditions (Hoisington et al., 1999; Brummer et al., 2011; Lippmann et al., 2019; Galluzzi et al., 2020; Letelier et al., 2020). Consequently, access to genetic resources and their evaluation for climate adaptation was identified as a topic of interest within the scientific community. Galluzzi et al. (2020) conducted a survey on the role of genetic resources in breeding for climate change in developing countries, finding a prevailing tendency among respondents to use advanced or elite germplasm as a source of genes to breed for drought tolerance. However, genetic diversity within elite germplasm may be low, and alleles related with adaptation to non-optimal growing conditions may have been lost during crop domestication and breeding. Breeders have started looking for genetic sources outside of modern cultivars in search of traits that may enable crops to tolerate prevailing or expected environmental conditions. Wild and domesticated accessions stored in gene banks around the world may contain genes related to crop tolerance to increased annual temperatures, reduced winter chill accumulation, reduced annual precipitation, prolonged drought events, and increased pest pressure that are not present in elite materials. Perez et al. (2020) evaluated old peach cultivars on the Canary Island La Palma, to look for germplasm adapted to warmer conditions, and Carvalho et al. (2017) evaluated ten traditional Portuguese grapevine varieties to identify genes associated with tolerance to abiotic stresses. In olive, a crop that is highly adapted to dry conditions, a collection of wild crop relatives was evaluated with microsatellite markers to determine suitable genotypes to be used as rootstocks to improve olive productivity (Diaz-Rueda et al., 2020a). Nevertheless, according to Galluzzi et al. (2020), the lack of knowledge on genetic resources and cultivar diversity in several species limits their use for accessing important traits for adaptation to climate change conditions.

Genetic resources in the form of cultivated crops are threatened by global warming, which may reduce the fitness of many wild species in their natural habitats (Lippmann et al., 2019). The loss of natural habitat and crop genetic diversity and the disappearance of native germplasm signify setbacks for breeding efforts for climate adaptation. Access to a wide range of genetic diversity is critical for the success of plant breeding agendas (Hoisington et al., 1999), highlighting the importance of strengthening policies to incentivize germplasm collection and conservation.

Although natural populations and germplasm collections may harbor genes that can confer adaptation benefits to commercial crops (Lobos and Hancock, 2015; Perez et al., 2020; Diaz-Rueda et al., 2020b), to facilitate their rapid adoption by breeding programs, genomic regions associated with traits of interest need to be mapped and characterized. Novel genomic technologies developed in recent years have provided new gene mapping techniques and allowed the development of molecular markers that can accelerate the efficient deployment of beneficial alleles to new cultivars (Lobos and Hancock, 2015; Iwata et al., 2016; Whitaker et al., 2020). For instance, because chilling requirements are becoming a limiting factor for cultivar adaptation in warming environments, scientists are working on identifying genes associated with bud dormancy and flowering time in temperate crops to elucidate the genetic control of these processes and develop new cultivars adapted to different climatic conditions (Castede et al., 2014; Romeu et al., 2014; Allard et al., 2016; Prudencio et al., 2018; Cantin et al., 2020; Prudencio et al., 2021; Zhang et al., 2022). New technologies have also enabled the identification of the genetic regions controlling traits such as flowering time (Ruiz et al., 2019; Branchereau et al., 2023) and bud dormancy (Allard et al., 2016; Prudencio et al., 2018, Prudencio et al., 2021), as well as traits related to temperature requirements for endodormancy release (Romeu et al., 2014). The identification of regions harboring genes related with the response of trees to environmental cues has facilitated the efficient use of molecular markers to reliably select genotypes that are better suited to particular climatic conditions.

As mentioned before, new pathogens will probably migrate to temperate regions, posing new challenges for disease management. In consequence, resistance to pests and diseases is one of the main objectives for breeding programs, and it will continue to play an important role along with the improvement of other phenotypic traits to overcome the challenges of climate change (Brummer et al., 2011; Lippmann et al., 2019). For a cultivar to be successful, adaptation and performance must be evaluated in multi-environment trials to assess the effects of genetic and environmental factors, as well as the effects of interactions between these factors on crop quality and productivity (Navas-Lopez et al., 2019; Farsi et al., 2023). Such trials are instrumental for identifying cultivars that produce reliably across environments or to select the best performers for specific conditions. In this context, the role of breeders is to identify the individuals with the highest breeding value given the local environment. Hence, local research organizations and universities, governments and private companies need to invest in local breeding programs to take timely actions in response to the needs of local environments and markets (Brummer et al., 2011; Galluzzi et al., 2020).

## 5 Conclusions and future direction

With the rapid expansion of climate change-related literature, systematic analyses can support researchers aiming to identify aspects that are well understood, topics that have remained unexplored and urgent questions that need to be addressed in the future.

Despite the possibility that we may have missed some pertinent articles due to methodological limitations, we believe that our key research topic analysis provides a good overview of the state of knowledge and constitutes a valuable resource that can help researchers from different disciplines identify critical knowledge gaps. Based on our systematic literature review, we find that several prominent topics have not or insufficiently been covered in the existing literature:

i. The recent rapid development and declining costs of molecular tools and computational capabilities have provided new opportunities to accelerate breeding of new cultivars adapted to extreme conditions. New approaches include high-throughput phenotyping and genotyping, genomic selection and predictive breeding driven by artificial intelligence. Efforts are still needed to adjust such technologies to the particular biological context of fruit and nut trees.
ii. The possible medium- or even long-term impacts of climate change on dormancy and phenology of temperate fruit trees remain unclear. To our knowledge, most studies have focused on immediate impacts, with little attention to tree responses during subsequent growing seasons. Knowledge about the trees’ ability to reset after unusually warm or dry seasons (as well as on the mechanisms involved) can greatly improve our understanding of factors that regulate dormancy during winter. Similar to developing new cultivars, such advances may also present opportunities for the development of effective adaptation strategies.
iii. After decades of stagnation, the validation, development and application of dormancy models has picked up speed during the time period we evaluated. Even though this work has led to some promising new avenues in modeling tree dormancy, it has also exposed glaring knowledge gaps. Prominent gaps are related to possible species- or cultivar-specific differences in chill (and heat) responses, to the precise nature of chill and heat accumulation dynamics, and to the validity and reliability of the models that are currently applied by scientists and practitioners.
iv. What remains conspicuously absent are dormancy models that are based on the current state of knowledge across the whole range of disciplines that have focused on dormancy research. Even though a comprehensive conceptual model of tree dormancy has been proposed (Fadon et al., 2020), this model still appears far from being turned into a computable model that could be used for predictions and for guiding further research on dormancy. Possibly, knowledge on tree dormancy is still too fragmented or the dormancy release process may be too complex to be reliably modeled at this point in time, but we remain hopeful that the coming decade(s) will deliver some progress in this space.
v. While Mediterranean climate regions have traditionally been ideal for temperate fruit crops, climate change related issues such as insufficient water availability for irrigation or deficient chill accumulation threaten production in these areas. The implementation of protective cover technologies has allowed for an expansion of production to cooler areas where drought and chill accumulation are not yet limiting production. However, these adaptation strategies cause significant microclimate variation with potential impacts on physiological processes and tree phenology. As the use of protective covers becomes more frequent, further research on the effects of such practices on tree nutrient and water demand is needed for designing management strategies that optimize yield and quality.
vi. Agroecological management can also be evaluated as an effective strategy to increase climate resilience. Although some studies found in this review addressed sustainable management practices such as non or minimum tillage, green manure, and soil amendments, the agroecological perspective involves a paradigm shift that considers the ecological equilibrium of the agroecosystem as a management strategy, focused on decreasing the dependence on external inputs. System that are guided by agroecological principles have been claimed to be comparatively resilient to climatic shocks and stresses, among other benefits (Altieri et al., 2015). More research focused on evaluating the effects of integrated agroecological management rather than assessing the effects of individual sustainable management practices is expected to support the transition towards systems that can adapt to current and future challenges.

Growing awareness of the effects that climate change may have on the future of our planet has triggered various local and international initiatives aimed at slowing down and hopefully reversing the buildup of greenhouse gases in the atmosphere. Here we report a diverse range of strategies focused on both mitigating the environmental impacts of fruit and nut production, and on adapting to current challenges stemming from changing weather patterns and extreme weather events that have become increasingly frequent and intense. However, the development and implementation of a set of strategies that secure the long-term sustainability of agricultural production and support farmers in adapting to future weather patterns may take a long time and require a multidisciplinary global effort. With varying vulnerability to climate change and diverse social and economic realities in different regions, policy changes along with technological development (e.g., breeding new cultivars, more efficient irrigation systems and water management and sustainable agricultural practices) need to be carefully crafted to meet local needs.

Finally, we strongly believe that global collaborative efforts that engage all actors of the production chain, including growers, distributors, retailers, and consumers, may optimize the chances of success in adapting the production of temperate fruit and nuts to the challenging future that lies ahead.

## Data availability statement

The original contributions presented in the study are included in the article/**Supplementary Material**. Further inquiries can be directed to the corresponding author.

## Author contributions

JO-M: Conceptualization, Data curation, Formal analysis, Investigation, Methodology, Visualization, Writing – original draft, Writing – review & editing. EF: Visualization, Writing – review & editing. LV: Conceptualization, Writing – review & editing. AR: Conceptualization, Funding acquisition, Writing – review & editing. EL: Visualization, Writing – review & editing. NC: Conceptualization, Formal analysis, Funding acquisition, Methodology, Supervision, Validation, Visualization, Writing – original draft, Writing – review & editing.
